# Omicron in Infants—Respiratory or Digestive Disease?

**DOI:** 10.3390/diagnostics13030421

**Published:** 2023-01-23

**Authors:** Anca Cristina Drăgănescu, Victor Daniel Miron, Oana Săndulescu, Anuţa Bilaşco, Anca Streinu-Cercel, Roxana Gabriela Sandu, Adrian Marinescu, Deniz Gunșahin, Karina Ioana Hoffmann, Daria Ștefana Horobeț, Daniela Pițigoi, Adrian Streinu-Cercel, Doina Anca Pleșca

**Affiliations:** 1 Faculty of Medicine, Carol Davila University of Medicine and Pharmacy, 050474 Bucharest, Romania; 2 National Institute for Infectious Diseases “Prof. Dr. Matei Balș”, 021105 Bucharest, Romania

**Keywords:** COVID-19, infants, children, Omicron, respiratory, digestive, SARS-CoV-2

## Abstract

The Omicron variant of SARS-CoV-2 has caused a large number of cases and hospitalizations in the pediatric population. Infants due to their age are susceptible to viral infections that may have a worse prognosis. Therefore, the aim of the current study has been to characterize the clinical features and the outcome of infants hospitalized with confirmed SARS-CoV-2 infection during the Omicron wave. We conducted a retrospective study of all consecutive infants hospitalized with symptomatic COVID-19 and no other co-infections, from January to September 2022 in one of the largest infectious diseases hospitals from Bucharest, Romania. A total of 613 infants were included in the analysis. The median age was 5 months (IQR: 3, 8 months). The clinical features were dominated by fever (96.4%), cough (64.8%) and loss of appetite (63.3%), and overall, respiratory symptoms were the most numerous (76.0%). Infants between 1-3 months old had a 1.5-fold increased risk of elevated alanine aminotransferase (ALT) values, and a longer length of hospitalization as compared to older infants. Infants between 7-9 months of age had 1.5-fold higher odds of loss of appetite, 1.7-fold more frequent cough and 1.6-fold more frequent digestive symptoms compared to infants in other age groups. The presence of digestive symptoms increased the probability of hepatic cytolysis (increased ALT) by 1.9-fold. Continued monitoring of COVID-19 among infants is very necessary, given the progressive character of SARS-CoV-2, in order to take correct and rapid therapeutic measures and to adapt to clinical changes driven by viral variant change.

## 1. Introduction

Infants are a particular group among children because of their age and specific anatomical and physiological characteristics [1,2]. The management of this type of patient during an acute infectious disease is based on a limited number of therapeutic resources and a careful follow-up of their clinical manifestations is necessary to prevent possible complications and an unfavorable course. Given the experiences with influenza viruses and respiratory syncytial virus for which infants are an at-risk group for hospitalization and potentially severe outcomes with respiratory failure [3,4,5], the onset of the COVID-19 pandemic has been regarded with concern [6] for this group of children.

However, somewhat unusually for a viral infection, children of all ages, including infants, have been significantly less affected than adults by COVID-19 since the beginning of the pandemic [7,8,9]. Factors hypothesized to have contributed to this initial trend included the non-pharmacological protective measures instituted, as well as the parental care for children [10,11], but different pathogenic mechanisms are also considered to have contributed to the different course of COVID-19 in children. Overall, children have had milder forms of the disease, with significantly lower rates of hospitalization and of severe disease compared to adults [7].

SARS-CoV-2 has undergone a number of changes in its viral structure so that the emergence of the Omicron variant caused an increase in the number of cases worldwide [12,13]. Omicron infection rates among children increased significantly, which has also resulted in an increase in hospitalizations for the pediatric population [13,14], but, fortunately, most cases have had a favorable outcome [13]. Although the first case of COVID-19 was identified in February 2020 [15], Romania experienced the largest wave of SARS-CoV-2 infections only after the first case of Omicron was identified [16] and the rate of confirmed cases of COVID-19 among children exceeded 10% [17].

The spectrum of clinical manifestations of COVID-19 in infants is highly heterogeneous, and each variant of SARS-CoV-2 has had a different impact on the clinical course in children [13,18]. Moreover, due to age, many subjective symptoms cannot be quantified and analyses on large groups of infants are needed to better characterize the impact of COVID-19 on this group of children. Therefore, we aimed at evaluating the spectrum of clinical symptoms of the omicron variant among infants hospitalized in one of the largest infectious disease hospitals in Romania.

## 2. Methods

We conducted a retrospective study among infants hospitalized with COVID-19 between 1 January and 30 September 2022 in the National Institute of Infectious Diseases “Prof. Dr. Matei Balș”, Bucharest, (NIID), with the aim of characterizing the clinical features and outcome of infants with SARS-CoV-2 infection, Omicron variant. NIID is the largest tertiary infectious disease hospital in Romania and during the pandemic it was the main care center for patients with SARS-CoV-2 infection in the capital and the nearby metropolitan areas.

We included in the study all infants (under 1 year of age) consecutively hospitalized during the study period (1 January–30 September 2022) with symptomatic SARS-CoV-2 infection, confirmed by RT-PCR from nasopharyngeal swabs. We excluded from the analysis asymptomatic infants, those with confirmed respiratory, digestive, urinary tract or systemic co-infections, those with incomplete data in the medical charts, and those who had been transferred from another hospital after more than 24 h of previous hospitalization. Infants who, during hospitalization, turned 1 year of age were considered eligible and included in the final analysis.

Each infant according to the clinical presentation at the time of the hospital admission received investigations such as multiplex RT-PCR of the respiratory tract, rapid stool antigen testing, stool culture, multiplex RT-PCR of stool, urine culture and blood culture. Any positive result in one of these investigations was considered co-infection with SARS-CoV-2 and these patients were excluded from the final analysis.

Data for each patient were extracted from the patient medical charts by teams formed by two of the authors of this article. Cough, rhinorrhea or nasal obstruction and dyspnea were considered respiratory symptoms, and vomiting, diarrhea or constipation were considered digestive symptoms. Given the age of the patients analyzed, symptoms such as headache, sore throat, fatigue, or other subjective manifestations could not be analyzed. Fever and loss of appetite were analyzed as separate symptoms from respiratory and digestive symptoms. Chest imaging assessment was performed in a small number of infants, so we have not reported these data.

In 2022, a total number of 683 infants were hospitalized in NIID with confirmed SARS-CoV-2 infection. After applying eligibility criteria in the final analysis, 613 (89.8%) of the infants were included.

Data analysis was performed using IBM SPSS Statistics for Windows, version 25 (IBM Corp., Armonk, NY, USA). For a p-value of less than 0.05, data were considered to be statistically significant. Since our continuous variables were not normally distributed, we present the median and the interquartile range (IQR: 25th–75th percentile). Comparative analysis between this type of data was done using the Mann–Whitney U test and the Kruskal–Wallis H test. For dichotomous variables we present frequencies and percentages and Chi-squared test values with odds ratios and 95%CI in the comparison analysis.

## 3. Results

### 3.1. General Data Analysis for the Whole Study Group On

A total of 613 infants were included in the analysis. The male sex was more numerous (n = 361, 58.9%), and the median age for the whole study group was 5 months (IQR: 3, 8 months), with a balanced distribution by age group (Table 1).

**Figure 1:**
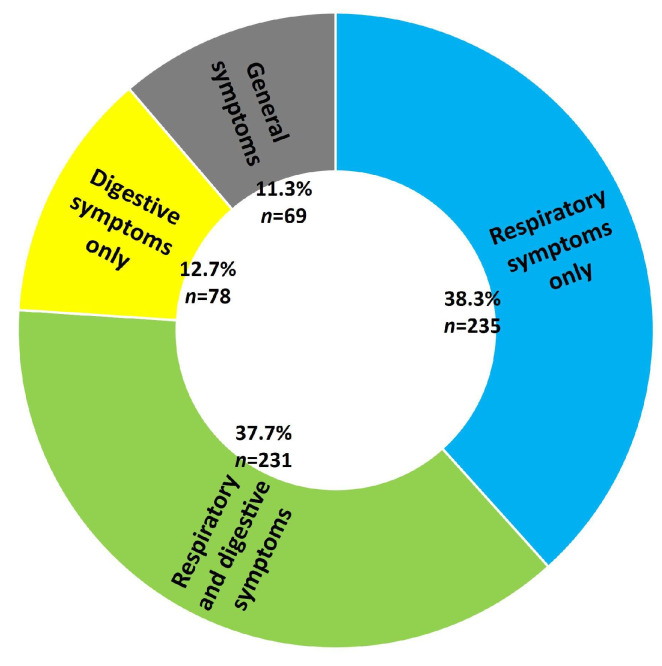
Distribution of symptom type in infants in the study.

The clinical presentation was dominated by fever (96.4%, n = 591), cough (64.8%, n = 397) and loss of appetite (63.3%, n = 388) (Table 1). Overall, respiratory symptoms were most common among infants with SARS-CoV-2 infection (76.0%, n = 466), and 11.3% (n = 69) had general manifestations only, such as fever and/or loss of appetite (Figure 1).

White blood cell (WBC) counts showed no significant patterns, with a median of 6800 cells/μL (IQR: 4900, 9500 cells/μL); 24.6% (n = 151) showed increased WBC and 7.3% (n = 45) decreased WBC. A high number of infants, 82.9% (n = 508), had anemia at the time of hospitalization with median hemoglobin values of 10.9 g/dL (IQR: 10.2, 11.7 g/dL). Aspartate aminotransferase (AST) and lactate dehydrogenase (LDH) values were elevated in 80.1% (n = 491) and 83.8% (n = 514) of cases, respectively. In contrast, alanine aminotransferase (ALT) was abnormally high in 32.1% (n = 191) of patients. Increases in interleukin-6 (IL-6) were identified in 96.7% (n = 88/91) of infants, with a median of 177.7 pg/mL (IQR: 57.5, 1333.5 pg/mL) (Table 2).

The majority of infants were hospitalized within 1 day (IQR: 0, 2 days) of symptom onset and the median length of hospitalization was 4 days (IQR: 3, 5 days). A total of 11.3% (n = 6) of infants had a chronic condition and 9.3% (n = 57) were premature. However, these risk factors were not associated with an increased length of hospitalization (*p* > 0.05). All infants had a favorable outcome, and none required admission to intensive care.

### 3.2. Data Analysis by Age Group

The analysis of data by age group is highlighted in Table 3. Infants between 1–3 months had a 5.5-fold increased risk of anemia (*p* < 0.001, χ^2^ = 27.07, OR = 5.5, 95%CI: 2.7–11.2) and a 1.5-fold increased risk of liver cytolysis with increased ALT values (*p* = 0.019, χ^2^ = 5.50, OR = 1.5, 95%CI: 1.1–2.2). Similarly, infants between 4–6 months had a 1.5-fold increased risk of having increased ALT values (*p* = 0.043, χ^2^ = 4.06, OR = 1.5, 95%CI: 1.1–2.2). The 7–9 months age group had a 1.2-fold increased risk of loss of appetite (*p* = 0.022, χ^2^ = 5.22, OR = 1.5, 95%CI: 1.1–2.4), a 1.7-fold higher risk of cough (*p* = 0.013, χ^2^ = 6.07, OR = 1.7, 95%CI: 1.1–2.5) and 1.6-fold higher risk of having digestive symptoms (*p* = 0.009, χ^2^ = 6.85, OR = 1.6, 95%CI: 1.1–2.4). In terms of laboratory parameters, there was a 2.1-fold increased risk of elevated AST values in this age group (*p* = 0.006, χ^2^ = 7.55, OR = 2.1, 95%CI: 1.2–3.6). For infants between 10–12 months there was a 1.9-fold higher risk of vomiting (*p* = 0.003, χ^2^ = 8.73, OR = 1.9, 95%CI: 1.2–3.1) and 1.7- and 2.1-fold higher risks of WBC (*p* = 0.020, χ^2^ = 5.35, OR = 1.7, 95%CI: 1.1–2.7) and C-reactive protein (*p* = 0.001, χ^2^ = 9.66, OR = 2.1, 95%CI: 1.3–3.3) increases, respectively.

**Figure 2:**
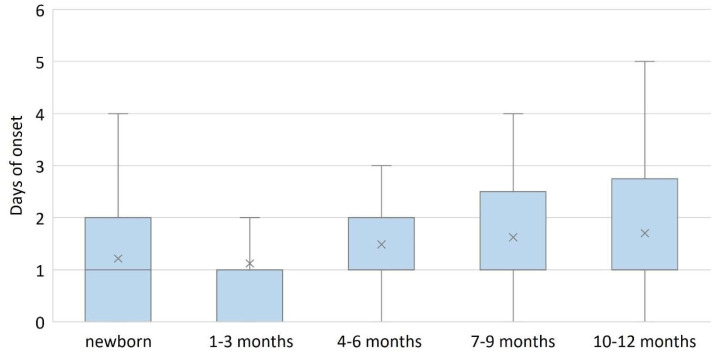
Number of days of onset of symptoms according to age group. Infants between 1–3 months presented to the hospital earliest after the onset of symptoms. In contrast, infants between 9–12 months presented the latest. Box and whiskers plot represent the 25th and 75th percentiles (box), the median (×) and the range (whiskers).

**Figure 3:**
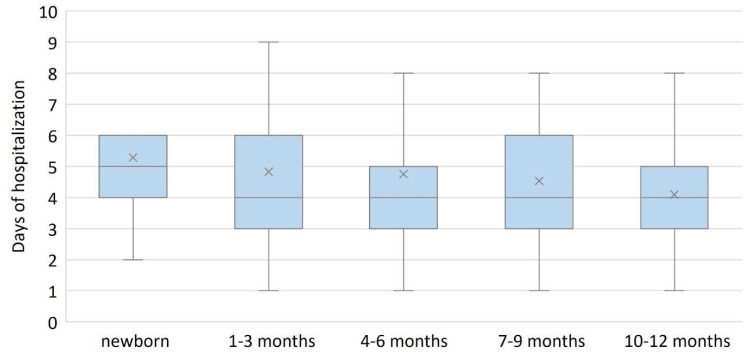
Number of days of hospitalization according to age group. The length of hospitalization was longer in infants between 1 and 3 months. Box and whiskers plot represent the 25th and 75th percentiles (box), the median (×) and the range (whiskers).

The infants in the 1-3 months group presented earliest to the hospital (1 day (IQR: 0, 1 days)), and those in the 10–12 months group presented latest (1 day, (IQR: 1, 2.75 days)), H(4) = 17.323, *p* = 0.002, Figure 2. Also, the length of hospitalization was the highest in the 1–3 months group (4 days, (IQR: 3, 6 days)), H(4) = 13.674, *p* = 0.008, Figure 3.

### 3.3. Analysis of Data by Type of Symptoms

The analysis of data by type of symptoms is presented in Table 4. The presence of combined respiratory and digestive symptoms was more common among males (*p* = 0.028, χ^2^ = 4.82, OR = 1.5, 95%CI: 1.1–2.0). In contrast, females were more likely to have digestive symptoms only (*p* = 0.028, χ^2^ = 4.82, OR = 1.5, 95%CI: 1.1–2.0). For infants in the 7–9 months age group, it was 1.9-fold more common (*p* = 0.001, χ^2^ = 10.72, OR = 1.9, 95%CI: 1.3–2.7) to have both respiratory and digestive manifestations during the COVID-19 episode as compared to the other age groups. ALT was significantly higher, 1.9-fold, among infants who experienced only digestive manifestations (*p* = 0.011, χ^2^ = 6.44, OR = 1.9, 95%CI: 1.1–3.0).

**Figure 4:**
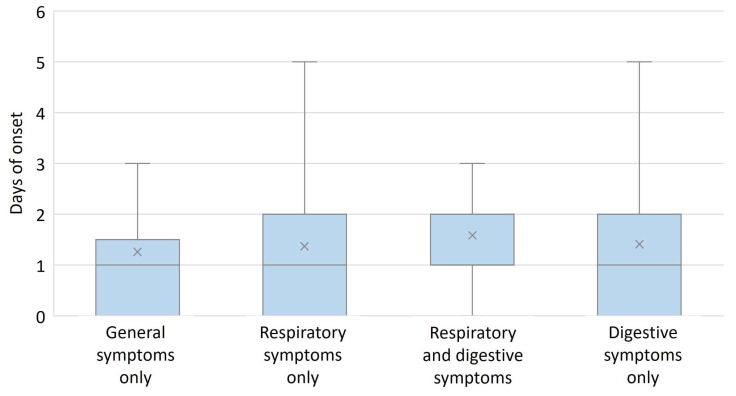
Number of days since onset of symptoms according to type of symptoms. The presence of general symptoms only (no digestive or respiratory symptoms) led to an earlier hospital presentation among infants. Box and whiskers plot represent the 25th and 75th percentiles (box), the median (×) and the range (whiskers).

**Figure 5:**
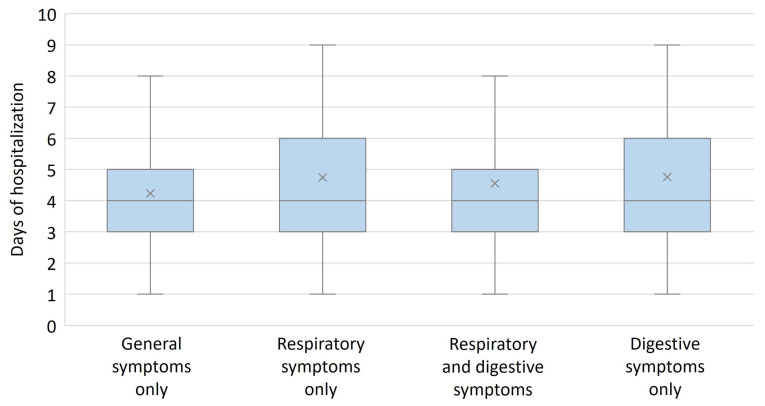
Number of days of hospitalization according to type of symptoms. The length of hospitalization was not influenced by the type of symptoms and was similar in all groups. Box and whiskers plot represent the 25th and 75th percentiles (box), the median (×) and the range (whiskers).

The presence of general manifestations only (fever and/or loss of appetite) led to an earlier hospital presentation in infants with COVID-19, H(3) = 11.679, p = 0.009, Figure 4. Length of hospital stay was not influenced by the type of symptoms presented by infants, H(3) = 4.002, *p* = 0.261, Figure 5.

## 4. Discussion

In the present study we have conducted an extensive analysis of the demographic, clinical and laboratory characteristics of SARS-CoV-2 Omicron variant infection among infants hospitalized in a major infectious disease hospital in Romania. The number of children hospitalized for COVID-19 increased with the emergence of the omicron variant (65.0% of all children hospitalized in NIID since the onset of the pandemic), and infants represented an age group that required close medical monitoring during their COVID-19 episode. This trend is consistent with national [17] and international reports [13,19,20]; for example, in the USA, by early 2022 the proportion of children hospitalized with COVID-19 had increased four-fold from previous waves [21]. In a meta-analysis of pediatric cases of SARS-CoV-2 infection in 2020, Bhuiyan et al. showed that infants represented 53% of all pediatric COVID-19 hospitalizations, but most were asymptomatic [22]. During the Delta variant circulation, epidemiological monitoring of SARS-CoV-2 infection showed an increase in pediatric cases and hospitalizations of infants, but these were associated with an increased incidence of COVID-19 in the adult population as well and were not due to increased Delta virulence in infants [23].

Fever (96.4%), cough (64.8%) and loss of appetite (63.3%) were the main symptoms of infants hospitalized with Omicron in our study. Overall, we showed that three out of four infants (76.0%) had a respiratory symptom and one out of two infants (49.6%) had a digestive symptom. Diarrhea was the main digestive manifestation in 37.5% of patients. In a report of 300 infants with COVID-19 from March to December 2020, fever and cough were also the two main symptoms present in different proportions, 77% and 40%, respectively [24]. In the same study, loss of appetite was reported in only 18% of infants, and digestive manifestations such as diarrhea (24%) and vomiting (10%) were also less frequent than in our study [24]. During the Delta variant, fever remained the main symptom in infants, but cough, rhinorrhea, diarrhea or vomiting were reported in lower percentages than in our study [13,23,25]. Both ours and most pediatric studies on COVID-19 highlighted the male sex as more likely to have symptomatic infection and hospitalization. In addition, we showed that female infants had a 1.7-fold higher risk of hospitalization when they had digestive manifestations only.

In the age group analysis, fever dominated in all age groups. Moreover, as significant clinical features we identified that loss of appetite, cough and digestive manifestations (of any type) were more common in those between 7–9 months of age, while vomiting was most common in infants between 10–12 months of age. We did not identify a similar analysis of COVID-19 manifestations by age group in existing field literature. Thus, this analysis is important in identifying clinical differences between infants, knowing that the rate of growth and development of infants is very rapid, and the therapeutic and management interventions of an infant are directly dependent on their age.

The clinical features of COVID-19 in children are similar to other viral infections, but their dominance varies widely by pediatric age group. Many symptoms are subjective and depend on children’s ability to describe them. In many case reports fever was the most common symptom at presentation, followed by cough, rhinorrhea and sore throat for the entire pediatric population up to 18 years of age [26,27,28,29,30]. Additionally, Zhou et al. showed that nasal congestion/rhinorrhea, sore throat, abdominal pain, and digestive manifestations were commonly seen but did not have a strong association with COVID-19 in children [31]. This is of interest, but the analysis was performed on a heterogeneous age group of 0–18 years, therefore we believe that narrow age group analyses are essential for a comprehensive characterization of SARS-CoV-2 infection in children. Infants are a special group that cannot express certain symptoms, therefore our study focused only on the analysis of symptoms that could be quantified by the parent/physician.

Blood counts did not change significantly, but the increase in WBCs (24.6%) and decrease in lymphocytes (13.7%) were seen in higher proportions than in other studies with non-Omicron variants [32,33]. A high number of infants had anemia, a mild form (82.9%), especially in the 1–3 months age group. This finding should be interpreted with caution, as it may not be due to SARS-CoV-2 infection alone, particularly given the fact that some infants experience physiological anemia in the first months of life. We identified high percentages of infants with mild increases in AST and LDH, compared with reports from other studies [32,33,34]. These data are of great interest because they show that in infants Omicron infection presents a systemic involvement, not being limited to the respiratory or digestive tract. ALT, as a sign of hepatic cytolysis, was elevated in fewer infants and was significantly associated with a younger age (1–3, and 4–6 months), and only with the presence of digestive symptoms. These findings highlights the need for monitoring liver enzymes in clinical practice in infants under 6 months of age presenting with digestive manifestations only. The presence of inflammatory syndrome was evident in most infants, but IL-6 elevations were significant in all age groups at higher values than for non-Omicron variants, as reported in the literature (177.7 vs. 120.36 ng/mL) [34].

Newborns and infants between 1–3 months of age presented more quickly to the hospital after the onset of symptoms, compared to other infants. In addition, only the presence of general signs (fever and/or loss of appetite) also determined earlier hospital presentation. These aspects can be explained by parental worries and fears related to the very young infant, and the uncertainty of a diagnosis, without other symptoms, only in the presence of fever. Moreover, it is well known that infants under 3 months of age with signs of acute illness are a pediatric emergency. It is therefore important for parents to keep in close contact with their pediatrician and/or general practitioner to identify early alarm signs.

The median length of hospital stay was 4 days, comparable to other published reports for the Omicron variant [24,33], and young infants (1–3 months) required longer monitoring compared with other infants.

Overall, in our analysis we have shown that Omicron in infants has a very diverse spectrum of clinical manifestations with predominantly respiratory, but also intricate respiratory–digestive manifestations. Thus, clinical diagnosis can be difficult to establish based on symptoms alone, and testing for SARS-CoV-2 should remain a standard practice in emergency departments for rapid and targeted epidemiological and therapeutic measures.

Our study has several limitations mainly represented by the retrospective nature of the data and the absence of long-term follow-up of infants included in the study. The large number of infants included in the analysis with SARS-CoV-2 infection, however, provides a comprehensive overview of the burden that the Omicron variant has had in this pediatric group. The first year of every child’s life is very important in physical, mental, and cognitive development. In this first year of life there are many stages of development, the newborn (0–28 days) being totally different from the young infant (1–3 months), and the latter being totally different from older infants (9–12 months). Infectious diseases among infants represent a big challenge, both for diagnosis (the clinical features are often atypical and vary according to the age groups mentioned above) and for treatment (each age group comes with certain treatment restrictions). If we analyze RSV or influenza viruses infection, we can see that infants are a group at risk of hospitalization and unfavorable outcomes. Therefore, we decided to perform an extensive analysis of infants hospitalized with Omicron. The Omicron variant is currently the dominant variant of SARS-CoV-2, and at the same time the variant that has caused the highest rates of morbidity and hospitalization among the pediatric population. We included a large number of infants in the study and performed an analysis of clinical and laboratory data. In addition, we conducted an analysis by age subgroups precisely to highlight the variability of these characteristics among infants. So far there are several reports on SARS-CoV-2 infection, including Omicron, among children, but none of them focus specifically on infants and age subgroups.

## 5. Conclusions

Infants are a pediatric group with very heterogeneous manifestations during SARS-CoV-2 infection, and these may be different even among them depending on age group. Age under 3 months was associated with earlier presentation to hospital and longer duration of hospitalization, and hepatic cytolysis was more common in infants with digestive manifestations only. Continued monitoring of COVID-19 among infants is highly necessary, given the evolving nature of SARS-CoV-2, in order to take accurate and rapid therapeutic and epidemiological measures, and to adapt to clinical changes driven by viral variant change.

## Figures and Tables

**Table 1:** Demographic and clinical characteristics for all infants included in the study.

Characteristic	Frequency, n	Percentage, %
Male sex	361	58.9
Age groups		
Newborn	14	2.3
1–3 months	182	29.7
4–6 months	160	26.1
7–9 months	149	24.3
10–12 months	108	17.6
Clinical features		
Fever	591	96.4
Loss of appetite	388	63.3
Respiratory symptoms	466	76.0
Cough	397	64.8
Rhinorrhea	295	48.1
Dyspnea	69	11.3
Digestive symptoms	309	49.6
Vomiting	148	24.1
Diarrhea	230	37.5
Constipation	21	3.4
Preterm	57	9.3
At least one chronic condition	69	11.3

**Table 2:** Laboratory findings in infants included in the study.

Laboratory Analysis	Results
WBC count, median (IQR)	6800 (4900, 9500) cells/μL
WBC increase, n (%)	151 (24.6)
WBC decrease, n (%)	45 (7.3)
Lymphocytes count, median (IQR)	3000 (1700, 5300) cells/μL
Lymphocytes decrease, n (%)	84 (13.7)
Hemoglobin, median (IQR)	10.9 (10.2, 11.7) g/dL
Anemia, n (%)	508 (82.9)
Platelets count, median (IQR)	278,000 (210,000, 356,000) cells/μL
Platelets increase, n (%)	11 (1.8)
Platelets decrease, n (%)	0 (0.0)
AST, median (IQR)	66 (53, 82) U/L
AST increase, n (%)	491 (80.1)
ALT, median (IQR)	32 (25, 45) U/L
ALT increase, n (%)	191 (31.2)
LDH, median (IQR)	350 (304, 406) U/L
LDH increase, n (%)	514 (83.8)
CRP, median (IQR) *	2.7 (0.9, 7.6) mg/L
CRP increase, n (%) *	182/509 (35.8)
IL-6, median (IQR) ^×^	177.7 (57.5, 1333.5) pg/mL
IL-6 increase, n (%) ^×^	88/91 (96.7)

WBC—white blood cells, ALT—alanine aminotransferase, AST—aspartate aminotransferase; LDH—lactate dehydrogenase; CRP—C-reactive protein; IL-6—interleukin 6; * Data available for 509 patients; ^×^ Data available for 91 patients.

**Table 3:** Data analysis according to age group.

Characteristics	Newborn N = 14	1–3 Months N = 182	4–6 Months N = 160	7–9 Months N = 149	10–12 Months N = 108	*p*-Value for Comparison between All Groups
Male sex, n (%)	10 (71.4)	93 (51.1)	96 (60.0)	96 (64.4)	66 (61.1)	0.105
Fever, n (%)	12 (85.7)	176 (96.7)	155 (96.9)	145 (97.3)	103 (95.4)	0.243
Loss of appetite, n (%)	11 (78.6)	109 (59.9)	93 (58.1)	106 (71.1)	69 (63.9)	0.093
Respiratory symptoms, n (%)	8 (57.1)	131 (72.0)	122 (76.3)	121 (81.2)	84 (77.8)	0.149
Cough, n (%)	3 (21.4)	106 (58.2)	111 (69.4)	109 (73.2) ^+^	68 (63.0)	**<0.001**
Rhinorrhea, n (%)	6 (42.9)	80 (44.0)	76 (47.5)	84 (56.4)	84 (45.4)	0.211
Dyspnea, n (%)	2 (14.3)	15 (8.2)	23 (14.4)	15 (10.1)	14 (13.0)	0.433
Digestive symptoms, n (%)	5 (35.7)	76 (41.8)	84 (52.5)	89 (59.7) ^+^	55 (50.9)	0.016
Vomiting, n (%)	2 (14.3)	20 (11.0)	39 (24.4)	49 (32.9)	38 (35.2) ^+^	**<0.001**
Diarrhea, n (%)	4 (28.6)	59 (32.4)	68 (42.5)	65 (43.6)	34 (31.5)	0.083
Constipation, n (%)	0 (0.0)	7 (3.8)	7 (4.4)	2 (1.3)	5 (4.6)	0.484
Preterm, n (%)	4 (28.6)	17 (9.3)	17 (10.6)	10 (6.7)	9 (8.3)	0.099
Chronic conditions, n (%)	1 (7.1)	19 (10.4)	22 (13.8)	13 (8.7)	14 (13.2)	0.622
WBC increase, n (%)	3 (21.4)	29 (15.9)	41 (25.6)	42 (28.2)	36 (33.3) ^+^	**0.011**
WBC decrease, n (%)	0 (0.0)	17 (9.3)	12 (7.5)	9 (6.0)	7 (6.5)	0.614
Lymphocytes decrease, n (%)	1 (7.1)	23 (12.6)	19 (11.9)	21 (14.1)	20 (18.5)	0.513
Anemia, n (%)	8 (57.1)	173 (95.1) ^+^	132 (82.5)	119 (79.9)	76 (70.4)	**<0.001**
AST increase, n (%)	8 (57.1)	128 (70.3)	133 (83.1)	131 (87.9) ^+^	91 (84.3)	**<0.001**
ALT increase, n (%)	3 (21.4)	69 (37.9) ^+^	60 (37.5) ^+^	37 (24.8)	22 (20.4)	**0.003**
LDH increase, n (%)	13 (92.9)	147 (80.8)	133 (83.1)	124 (83.2)	97 (89.9)	0.281
CRP increase *, n (%)	1/10 (10.0)	25/140 (17.9)	55/133 (41.4)	56/136 (41.2)	45/90 (50.0) ^+^	**<0.001**
IL-6 increase ^×^, n (%)	1/1 (100)	18/18 (100)	21/21 (100)	30/32 (93.8)	18/19 (94.7)	0.656

WBC—white blood cells, ALT—alanine aminotransferase, AST—aspartate aminotransferase; LDH—lactate dehydrogenase; CRP—C-reactive protein; IL-6—interleukin 6; * Data available for 509 patients; ^×^ Data available for 91 patients; ^+^ Age group with statistical significance; In bold, data with statistical significance by comparing characteristics for all groups by χ^2^(4).

**Table 4:** Analysis of data according to the type of symptoms.

Characteristics	General Symptoms Only, N = 69	Respiratory Symptoms Only, N = 235	Respiratory and Digestive Symptoms, N = 231	Digestive Symptoms Only, N = 78	*p*-Value
Male	35 (50.7)	140 (59.6)	149 (64.5) ^+^	37 (47.4)	**0.027**
Female	34 (49.3)	95 (40.4)	82 (35.5)	41 (52.6) ^+^	
Newborn	3 (4.3)	6 (2.6)	2 (0.9)	3 (3.8)	**0.049**
1–3 months	27 (39.1)	79 (33.6)	52 (22.5)	24 (29.7)	
4–6 months	15 (21.7)	61 (26.0)	61 (26.4)	23 (29.5)	
7–9 months	12 (17.4)	48 (20.4)	73 (31.6) ^+^	16 (20.5)	
10–12 months	12 (17.4)	41 (17.4)	43 (18.6)	12 (15.4)	
Preterm	2 (2.9)	24 (10.2)	28 (12.1)	3 (3.8)	0.056
At least one chronic condition	5 (7.2)	32 (13.6)	25 (10.8)	7 (9.0)	0.412
WBC increase	14 (20.3)	70 (29.8)	54 (23.4)	13 (16.7)	0.074
WBC decrease	5 (7.2)	17 (7.2)	16 (6.9)	7 (9.0)	0.947
Lymphocytes decrease	12 (17.4)	29 (12.3)	30 (13.0)	13 (16.7)	0.606
Anemia	60 (87.0)	187 (79.6)	193 (83.5)	68 (87.2)	0.295
AST increase	58 (84.1)	178 (75.7)	194 (84.0)	61 (78.2)	0.120
ALT increase	18 (26.1)	61 (26.0)	78 (34.2)	34 (42.3) ^+^	**0.044**
LDH increase	54 (78.3)	198 (84.3)	198 (85.7)	64 (82.1)	0.494
CRP increase *	20/54 (37.0)	74/193 (38.3)	71/195 (36.4)	17/67 (25.4)	0.286
IL-6 increase ^×^	7/7 (100)	42/44 (95.5)	32/33 (97.0)	7/7 (100)	0.873

WBC—white blood cells, ALT—alanine aminotransferase, AST—aspartate aminotransferase; LDH—lactate dehydrogenase; CRP—C-reactive protein; IL-6—interleukin 6; * Data available for 509 patients; ^×^ Data available for 91 patients; ^+^ Symptoms group with statistical significance; In bold, data with statistical significance by comparing characteristics for all groups by χ^2^(3), and χ^2^(12) for age groups.

## Data Availability

The datasets generated and analysed during the current study are available from the corresponding author upon reasonable request.
